# Missing the egocentric spatial reference: a blank on the map

**DOI:** 10.12688/f1000research.13675.1

**Published:** 2018-02-09

**Authors:** Maria Concetta Miniaci, Elvira De Leonibus

**Affiliations:** 1 Department of Pharmacy , School of Medicine, University of Naples Federico II, Naples, Italy; 2 Institute of Genetics and Biophysics (IGB) , National Research Council, Naples, Italy; 3 Telethon Institute of Genetics and Medicine, Telethon Foundation, Pozzuoli, Italy

**Keywords:** egocentric navigation, posterior parietal cortex, striatum, aging, Alzheimer’s disease, autism.

## Abstract

Egocentric (self-centered) and allocentric (viewpoint independent) representations of space are essential for spatial navigation and wayfinding. Deficits in spatial memory come with age-related cognitive decline, are marked in mild cognitive impairment (MCI) and Alzheimer’s disease (AD), and are associated with cognitive deficits in autism. In most of these disorders, a change in the brain areas engaged in the spatial reference system processing has been documented. However, the spatial memory deficits observed during physiological and pathological aging are quite different. While patients with AD and MCI have a general spatial navigation impairment in both allocentric and egocentric strategies, healthy older adults are particularly limited in the allocentric navigation, but they can still count on egocentric navigation strategy to solve spatial tasks. Therefore, specific navigational tests should be considered for differential diagnosis between healthy and pathological aging conditions. Finally, more research is still needed to better understand the spatial abilities of autistic individuals.

## Introduction

Successful navigation is a fundamental cognitive function that is crucial for survival. Humans, like other animals, must learn about the layout of their environments to return home, or move between known locations. The spatial reference frame used to locate positions and directions in a complex environment are commonly divided into two main systems: egocentric (subject-centered) and allocentric (object-centered) ^1, 2^. During navigation, information in both egocentric and allocentric reference frames can be integrated to provide a coherent representation of the environment and proper orientation.

**Figure 1.**
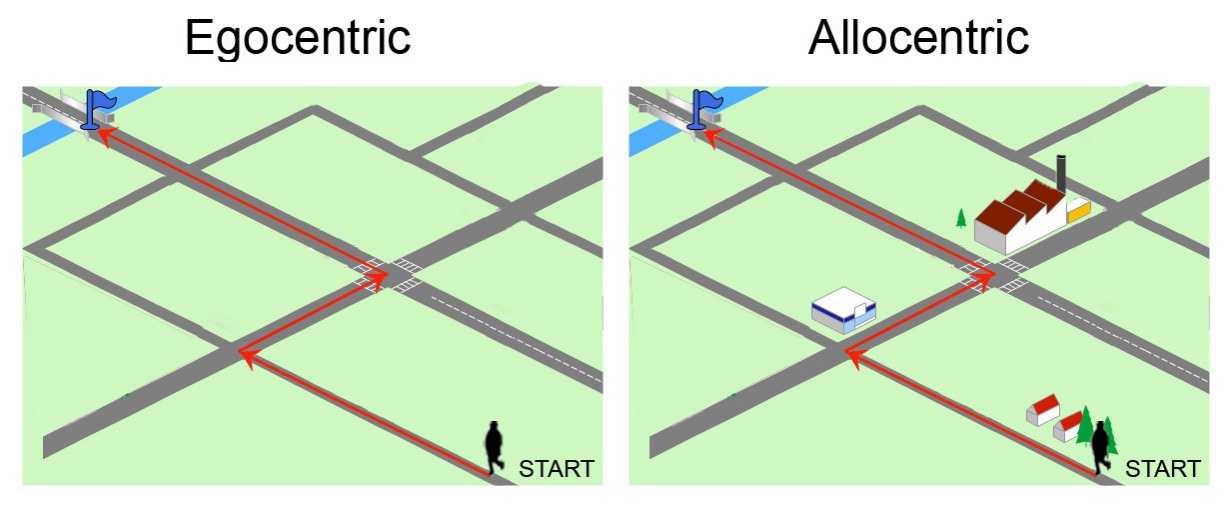
To reach the goal (bridge on the river), the subject can reproduce a sequence of left-right body turns (egocentric strategy) or use environmental cues (allocentric strategy).

Egocentric frames define spatial positions using the body, or a specific part of the body (head or trunk) as a point of reference ( Figure 1) ^3– 5^. Allocentric reference frame codes the position of the target relative to surrounding visual cues or landmarks and their spatial relationships, independently of the observer's current position ^6^. Such information can be used to build a “cognitive map”, a sort of internal representation of the environment ^7^.

Typically, the egocentric strategy relies upon kinesthetic and vestibular sensory information as well as motor command efferent copies, and optokinetic ﬂow information as the subject moves past surrounding objects ^8^. Egocentric learning ability has been demonstrated in paradigms in which animals must repeat a sequence of responses or movements toward a target, e.g., turning to the left, or reaching a fixed goal from a fixed starting point. Egocentric spatial orientation is likely to occur in the absence of external allothetic visual cues—e.g., in total darkness or in water maze with high and opaque walls that do not allow the use of extramaze cues. However, learning occurs relatively slowly in the water maze in darkness ^9, 10^, due to cumulative errors of the vestibular system, which are not corrected by visual inputs ^11^. Egocentric navigation is also associated with path integration, a strategy of spatial navigation that uses vestibular and proprioceptive cues generated during locomotion to keep track of position relative to a known starting point ^12^. Path integration or dead reckoning is, for example, used by foraging animals, such as desert ants and honeybees, to search for food along novel routes extending hundreds of meters ^13^. After reaching the site, those animals show an impressive level of accuracy at returning back to the nest using only the idiothetic cues generated by their movements. Environmental allothetic cues can be used to correct heading direction. Path integration can also be assessed in human subjects walking blindfolded to a previously seen target or asking them to estimate the distance and direction traveled while walking blindfolded ^14^.

## Neuronal basis of egocentric navigation

**Figure 2.**
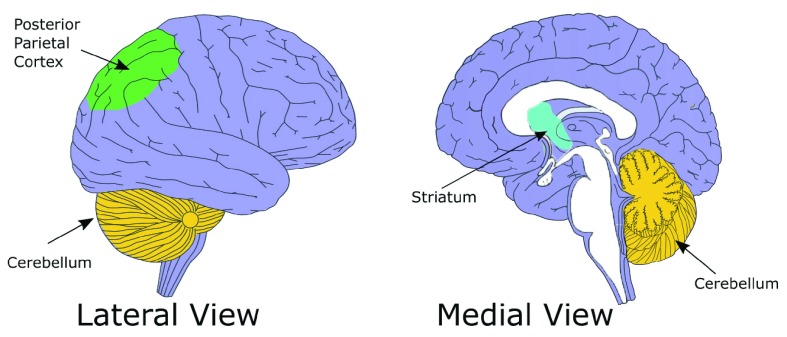
Brain regions involved in egocentric spatial representation.

Behavioral and brain imaging studies indicate that egocentric and allocentric strategies are mediated by a different cognitive-neural system, with some degree of overlapping. Egocentric-based navigation relies mainly on the posterior parietal cortex (PPC; Figure 2) whereas hippocampus and parahippocampal cortex are crucial for allocentric navigation ^15, 16^. The activity in the caudated nucleus has also been associated with egocentric tasks requiring a response strategy, such as following a well-learned route in a virtual city ^17^.

### Role of the posterior parietal cortex

The importance of PPC in egocentric navigation has been demonstrated by lesion studies in rodents and humans as well as by functional MRI (fMRI) studies in healthy subjects. Rogers and Kesner ^18^ have found that rats with selective lesions of the right posterior parietal cortex were impaired in the acquisition of the egocentric version of the water maze task, in the absence of extramaze cues, but had no difficulty in learning the allocentric version of the task, when extramaze cues were available. Rats with PPC lesions are also strongly impaired in path integration-based navigation under conditions in which visual inputs are irrelevant ^19^. For example, they are unable to locate the escape platform in a water maze when tested in complete darkness or to return directly to the refuge after searching for a randomly located food reward on a circular arena surrounded by a curtain, suggesting that the PPC plays an important role in idiothetic processing during path integration. Furthermore, single-unit recording data have demonstrated that a substantial fraction of cells in PPC of rats responds selectively to head orientation or to locomotion such as forward motion to the left or right ^20– 22^.

The involvement of PPC in egocentric spatial processing has also been observed in humans as they navigated to goal destinations in the virtual simulation of London ^23^. Analysis of the fMRI data revealed that the activity in bilateral PPC was significantly correlated with the egocentric direction to the goal. Additional evidence in support of a role of PPC in egocentric based spatial navigation comes from patients with PPC damage following cerebral infarction or hemorrhage in the right hemisphere ^24, 25^. Lesion of PPC can result in a contralateral neglect i.e. inattention to objects and space on the body opposite to the brain damage. Patients with neglect lose the ability to represent the location of objects (and landmarks) with respect to the self, though the landmarks are still recognizable. Indeed, they can get lost in their homes as they ignore left-hand turns or doorway. Neglect of representational space has been clearly described by Bisiach and Luzzatti ^26^. They asked patients with left-sided neglect to recall the layout of the Piazza del Duomo in Milan, a place very familiar to them. When imagining themselves facing the cathedral in the middle of the piazza, such patients neglected the left side of the piazza, recalling only the right side; however, when asked to view the piazza from the opposite end, they recalled the previously neglected buildings. An impairment of egocentric processing of remote spatial memory has also been observed in patients with focal lesion of PPC without clinical signs of neglect ^25^. In particular, when examined on mental navigation tasks in a very familiar environment (i.e., downtown Toronto), such patients were impaired on tasks that involved egocentric mental views of places such as describing an efficient route from one specific Toronto landmark to another. However, they showed preserved allocentric knowledge of the same environment, as they were able to indicate the location of landmarks on a map of Toronto or draw a map of the streets of Toronto.

Anatomical studies support the important role of PPC in processing egocentric spatial information ^27^. Indeed, PPC receives and integrates signals from cortical areas representing all main sensory modalities, such as vision, audition, proprioceptive, and vestibular. In addition, it is connected to cortical regions involved in goal-directed behavior such as the orbitofrontal, and medial prefrontal cortices and the striatum ^28^. The parietal cortex is also reciprocally connected with the hippocampal formation via the retrosplenial and the postrhinal cortex ^29^.

### Role of the striatal complex

Potegal ^30^ was the first to suggest that the striatal complex is involved in processing egocentric information; he based his hypothesis on the observation that patients with Huntington’s disease, which are affected by neurodegeneration of striatal neurons, were impaired when using the egocentric frame of reference.

The striatal complex is part of the basal ganglia nuclei, which receive information from the whole cortical mantle ( Figure 2). It was initially thought that these nuclei were selectively involved in the movement control, but more recent evidence clearly indicates that the basal ganglia, and in particular the striatum, also have important cognitive functions, including learning and memory ^31– 36^. Neuroanatomical evidence suggests that the striatal complex can be further distinguished in at least three different sub-regions including the dorsolateral and the dorsomedial striatum, corresponding to the putamen and the caudate in non-human and human primates, respectively, and the nucleus accumbens, which is located in the ventral striatum ^37^. The role of these different components in spatial navigation seems to follow lateromedial and dorsoventral gradient. The dorsolateral striatum is involved in all forms of egocentric navigation strategies with little involvement in allocentric navigation ^32, 33, 38– 43^. These findings are in line with neuroanatomical evidence showing that this part of the striatal complex receives dense projections from the PPC and the dorsolateral prefrontal cortex, as well as from the vestibular system, which is crucial for egocentric spatial information processing ^44– 46^. Both the medial and the ventral striatum are involved in egocentric spatial processing. However, these brain regions seem to be recruited in egocentric spatial tasks only when complex visuospatial tasks require a flexible updating system, like locating the position of a displaced object based on its position related to the subject body axes ^33^. The medio-ventral striatum receives a direct input from the hippocampal formation ^47, 48^ and has been particularly implicated in allocentric spatial information processing ^33, 49^.

The role of the dorsal striatum in egocentric spatial navigation has been attributed to its specific contribution to the formation of stimulus-response based habit learning ^50^; however, more recent evidence in rodents using one-trial learning tasks, such as the identification of a displaced object based on the subject position, suggests that habit learning and egocentric spatial information processing are two distinct functions involving the dorsal striatum ^32, 33^. This dissociation has been confirmed using an outcome evaluation protocol; habit responses are, by definition, controlled by the releasing stimulus, and not by the outcome. For example, if the subject always takes the same route from home to work, it may happen that the subject continues to take the same route for days even after the goal has been changed (such as moving to another place). This situation has been modeled in rodent studies, applying an outcome devaluation protocol after a maze learning. Egocentric strategy is initially used to navigate in relatively constant environments in a flexible manner ^35^; with increasing practice, the egocentric responses become habitual and resistant to changes in the environment and in the outcome value. Indeed, animals continued to turn right (or left) after learning even if their starting position has been changed by 180° or the food they obtained when turning right induced malaise. Furthermore, in the same study it was shown that deactivation of the dorsomedial striatum and the dorsolateral striatum mimicked and abolished, respectively, the effects of practice in the shift from egocentric to habit learning ^35^.

### Cerebellum and egocentric based motor sequence

Functional neuroimaging studies in humans have demonstrated that the cerebellum is activated during virtual navigation tasks that can be solved using either sequence-based strategy or place-based strategy ^51^. However, the two strategies appeared to involve different cerebellar lobules and cortical areas: place-based responses revealed the activation of the left cerebellar lobule VIIA Crus I, the right hippocampus and the medial parietal cortex, whereas for sequence-based response, right lobule VIIA Crus I, left hippocampus and medial prefrontal cortex medial were coactivated and functionally connected.

Experiments carried out in transgenic mouse model with selective inhibition of protein kinase C inhibitor (PKCI) in Purkinje cells, have brought new insights regarding the role of cerebellum in spatial navigation ^52, 53^. L7-PKCI transgenic mice lack parallel fiber–Purkinje cell long-term depression (LTD), which is considered the main neural correlate of cerebellar motor learning ^54– 56^. Interestingly, L7-PKCI mice exhibited disrupted hippocampal place cell properties when forced to use self-motion cues, such as navigation in total darkness. Consistently with their hippocampal place cell alteration, L7-PKCI mice were unable to navigate efficiently toward a goal in the dark, suggesting that the cerebellum may shape hippocampal activity during spatial navigation. The main hypothesis is that the cerebellum contributes to the formation of spatial representation by combining vestibular with proprioceptive inputs to generate appropriate information about body location in space ^57– 59^. The cerebellum appears to also be connected with the posterior parietal cortex, that encodes self-motion and acceleration ^22^. The interaction between PPC and cerebellar lobules has been shown in humans and monkeys and is believed to play an important role in planning and execution of navigation behavior.

## Egocentric navigation in childhood and aging

Studies on spatial abilities in childhood suggest that infants use mainly an egocentric reference system and that a gradual ability to use allocentric representations is acquired with age. Egocentric representation is considered a more elementary mean of representing the location of an object than an allocentric representation and, therefore, is already present early ^60, 61^. Acredolo and Evans ^62^ found that 6-month-old infants, trained to anticipate the appearance of a face at either their left or right and then turned around, continued to look in the same egocentric direction after they were rotated to the opposite side of the room. The correct use of allocentric representations develops progressively with increasing age. A further study carried out in children of 5, 7, and 10-year-olds has demonstrated that a high percentage of children spontaneously used the egocentric strategy on the virtual reality adaptation of the StarMaze task, reproducing the same sequence of body turns during the probe trials as during the training trials ^63^. The allocentric strategy, based on landmark guidance, was spontaneously used to solve the task in a few percentage of children at 7 and 10 years, but not at 5 years of age. However, when the allocentric strategy was imposed, the 5- year-olds were able to use allocentric behavior but their performance was below that of the 10-year-olds.

The shift from egocentric to allocentric strategy preference with increasing age is consistent with the heterogeneity in developmental trajectories of subcortical and cortical structures; decrease of subcortical and increase of cortical gray matter volumes have been described after age 7 ^64^.

After 60 years of age, there is a clear decline in spatial navigation abilities ^65^. Such decline is often related to functional changes of the posteromedial, the medial-temporal and the frontal areas ^66, 67^. Older adults present spatial navigation deficits particularly in allocentric navigation, being less effective in forming and using the cognitive maps when examined in both virtual and real-life versions of the human Morris maze ^68, 69^. On the other hand, the egocentric spatial navigation and learning is preserved in older age. The same results have been confirmed in studies testing older adults (71–84 years old) in a real-space human analog of the Morris Water Maze ^70^.

Behavioral studies on rodents have confirmed the effect of aging on the spatial task performance requiring the use of allocentric strategy ^71^. For example, aged mice were impaired in the water maze task in which they must remember the location of a submerged platform in relation to a series of extra-maze or distal cues, with the position of the starting point changing from trial to trial ^72^. However, the aged mice performed correctly the egocentric spatial task in a T-maze, remembering a sequence of movements (such as rotation to the left). These results suggest that aging can affect the allocentric and egocentric processing of spatial information in a different way.

These findings are in line with an increased sensitivity of brain regions of the medial temporal lobe, including the hippocampus, to the insult of aging and associated neurodegenerative disorders such as AD.

## Egocentric spatial deficits in developmental disorders: the case of autism

Based on neuropsychological literature showing that the use egocentric develops before allocentric spatial strategies ^73^, altered egocentric spatial ability can be considered an early sign of neuro- and psychiatric developmental disorders. This issue is generally little explored. However, some studies have addressed egocentric spatial information processing in Autism spectrum disorder (ASD). ASD is a multifactorial neurodevelopmental disorder that can also be secondary to genetic syndromes, including DiGeorge syndrome, Optiz syndrome or Fragile X syndrome ^74^. ASD manifests with impaired eye contact and communication skills, as well as impaired social interaction ^75^. Furthermore, children with autism often present stereotyped behavior and self-injury. Visuo-spatial abilities in subjects with autism have been investigated. Although in one study, no impairment was observed ^76^, more recent studies suggested that autistic adults show impaired performance in egocentric spatial tasks, when they have to use their body as reference frame ^77, 78^.

These deficits have never been directly associated to altered brain activation patterns. Interestingly, structural alteration of subcortical regions has been widely identified in autistic children. For example, the largest brain morphometric study in ASD to date shows that autism is associated with smaller subcortical volumes of the pallidum and putamen. Cerebellar cell loss and atrophy has also revealed in ASD children ^79– 83^.

Impaired subcortical functions in animal models of autism have been mainly associated to social behavior impairment, with little attention to the possible effects of spatial information processing. Behavioral investigation of MID1-null mouse model of Opitz G/BBB syndrome (OS), a genetic disorder characterized by mental retardation and brain abnormalities such as hypoplasia of the anterior cerebellar vermis, has demonstrated that these mice can promptly learn to locate the food in a T-maze if the position of the food remains constant relatively to extra-maze cues (allocentric strategy), but they are impaired if the position of the food is anchored to the animal position in the maze (egocentric strategy) ^84^. Impairment of egocentric spatial information processing has been also observed in children with Williams syndrome (WS), a neurodevelopmental disorder resulting from a hemizygous microdeletion of ~25 genes on chromosome 7q11.23 ^73, 85^.

Egocentric spatial abilities impairment correlates with altered social performance in ASD. Therefore, understanding the nature and the neuronal correlates of egocentric spatial abilities in ASD might be relevant to shed light on the nature of other cognitive and social deficits characterizing ASD, as evidenced by studies linking spatial and social cognitive abilities in ASD ^86, 87^.

## Egocentric impairments deficits in Alzheimer's disease

Alzheimer's disease (AD) is a neurodegenerative disorder characterized by the accumulation of amyloid plaques and neurofibrillary tangle accompanied by neuronal loss ^88^. In the early phase of the disease, most patients show cognitive impairments, such as memory deficits, language impairment, poor judgment and decision making ^89– 91^. As the disease progresses, reasoning, visual-spatial skills, and sensory processing become increasingly affected. The cognitive impairment in patients with AD is closely associated with the progressive degeneration of medial temporal lobe, including hippocampal formation and entorhinal cortex, frontal and parietal cortex and the basal forebrain ^92, 93^. Spatial navigation deficits, such as becoming disoriented or feeling lost in familiar places, represent the first sign of AD, and have, therefore, an important clinical utility for the early detection of AD ^94, 95^. Several studies in recent years have focused on spatial deficits in patients with amnestic Mild Cognitive Impairment (aMCI), which is considered an intermediate stage between the expected cognitive deficit of normal aging and the serious decline of dementia. Patients with amnestic MCI, especially those with the hippocampal type of amnestic syndrome, are at very high risk of AD. The conversion rate from MCI to dementia range is about 10% per year; in contrast to conversion rate from healthy elderly subjects to dementia which is about 1–2 percent per year ^96^. Interestingly, MCI patients are impaired in both allocentric and egocentric navigation, as documented by a series of study on virtual as well as real navigation. In one of the first studies, Hort and collaborators ^97^ tested a group of AD and aMCI patients on a goal-directed navigation task within a circular arena. The goal was invisible and could be identified either by its position relative to the starting position (egocentric subtest) or relative to cues on the wall (allocentric subtest). Each subtest began with an overhead view of the arena on a computer monitor. Both AD and aMCI were similarly impaired in all types of experiments, suggesting that spatial navigation impairment is not limited to AD, but begins to decline earlier in MCI. To understand the neural mechanisms behind spatial navigation of AD, Weniger and collaborators ^98^ examined a group of patients with aMCI on two virtual reality tasks, a virtual park containing several landmarks (allocentric memory) and a landmark-free virtual maze (egocentric memory) while undergoing an fMRI scan. They showed that aMCI patients were significantly impaired on both virtual reality tests, getting more frequently lost than controls and being unable to find a navigation strategy to learn the maze. Importantly, the allocentric and egocentric memory of aMCI patients were associated with size reduction of hippocampus, precuneus, and right-sided parietal cortex. These results have been recently confirmed by Boccia *et al.* ^99^. In their studies, patients with aMCI were challenged on an intensive learning paradigm, during which participants were shown two paths using either video clips or maps of a real city. The video clips were used to encourage participants to develop an egocentric representation of the city, whereas the maps were used to encourage them to develop an allocentric representation. After learning, they were asked to retrieve each of these paths, using an allocentric or egocentric frame of reference, while undergoing fMRI scan. Behavioral results indicated that aMCI showed a reduction in the rate of learning on the allocentric task. In addition, patients with aMCI showed a selective impairment in retrieving topographical memory using an egocentric perspective. Imaging data suggest that this general decline was correlated with hypoactivation of the brain areas generally involved in spatial navigation, e.g. medial temporal lobe structures, angular gyrus, and orbitofrontal cortex.

Furthermore, there is a strong evidence that patients with mild AD and aMCI are also impaired on path integration task, in which they are required to return to the starting point following an enclosed triangle pathway with a mask over their eyes ^100^. Such deficit appeared to be correlated to a reduced size of hippocampus, entorhinal and parietal cortices.

These findings suggest that neuropsychological screening designed to assess navigational deficit may represent an important tool for detecting the prodromal symptoms of aMCI and early stage of AD, and therefore, allow appropriate diagnosis and intervention.

## Conclusions

Many interesting points emerge from spatial navigation studies presented in this review. First of all, it appears that, unlike allocentric, the egocentric spatial strategy is quite preserved under physiological aging. But there are conditions such as AD in which egocentric ability is also impaired and this determines devastating effects on spatial navigation so that individual get lost even in a familiar environment. Secondly, the egocentric spatial deficits in brain disorders such as AD are often correlated with a reduced size of brain areas involved in egocentric information processing. Overall, these findings suggest that neuropsychological screening designed to assess navigational deficit may represent an important approach for detecting neurological and psychiatric disease progression, thus allowing an appropriate intervention.
