# Supplementary material for: Missing the egocentric spatial reference: a blank on the map
Source: F1000Res. 2018 Feb 9;7:168. [Version 1] doi: 10.12688/f1000research.13675.1 (PMC5840622; doi:10.12688/f1000research.13675.1)
Supplement: Peer review history

# Peer review: Referee response for version 1

**DOI:** 10.5256/f1000research.14855.r30709

**Published:** 2018-03-05

**Authors:** Silvia Middei

**Affiliations:** 1 Department of Experimental Neurology, Santa Lucia Foundation, Rome, Italy

This is an interesting review paper on spatial reference abilities under physiological and pathological conditions. The manuscript is well written and clear, also it draws relevant, though in some instances not novel, conclusions. I fully support the publication of this paper, and I only have a couple of minor comments. First, I agree with the authors about the possibility to use spatial tasks for a pre-clinical screening of people at risk for AD. For this reason, I would suggest to also include in the discussion the (few) available studies that investigated the relationship between spatial navigation deficits and markers of AD, including for instance CSF levels of Abeta (see studies from Head and collaborators) or amyloid PET scan. Second, the sentence *'egocentric develops before allocentric spatial strategies' *(and hence) 'altered *egocentric abilities... can be considered an early sign*' looks a bit confounding to me. If so, one would expect that deficits in allocentric spatial strategies may emerge after the ones in egocentric ones in ASD, which is not the case in the studies reported here. The general view of the paragraph is correct, then I would only suggest to re-phrase the first sentence.

I have read this submission. I believe that I have an appropriate level of expertise to confirm that it is of an acceptable scientific standard.

## References

[1] AllisonSFaganAMorrisJHeadD: Spatial Navigation in Preclinical Alzheimer’s Disease. *Journal of Alzheimer's Disease*.2016;52(1) : 10.3233/JAD-150855 77-90 10.3233/JAD-150855 PMC509181326967209

# Peer review: Referee response for version 1

**DOI:** 10.5256/f1000research.14855.r31042

**Published:** 2018-03-05

**Authors:** Sergio Chieffi

**Affiliations:** 1 Department of Experimental Medicine, University of Campania Luigi Vanvitelli , Naples, Italy

In this review, Miniaci and De Leonibus present a concise and balanced overview of the egocentric-based spatial navigation. In the first part, the authors focus on the brain areas mainly involved in egocentric information processing, such as the posterior parietal cortex and the striatum. Then, they review a series of studies showing that the egocentric spatial abilities are impaired in developmental disorders like autism, as well as under pathological aging conditions, such as those of Alzheimer's disease.

 The authors provide a critical view of the summarized literature leaving also some important questions open. For example, they realize how little attention has been paid to the study of spatial processing in autism and associated brain activation patterns. Indeed, understanding the nature and the neuronal correlates of spatial processing in autism can shed light on the nature of cognitive and social deficits that characterize the autism spectrum disorder. In addition, neuropsychological screening designed to assess navigational deficit may represent an important tool for detecting the prodromal symptoms of amnestic mild cognitive impairment and early stage of Alzheimer’s disease. Overall, the paper is well written and the figures are clearly presented.

 My recommendation to the authors is to improve the conclusions about the relevance of the egocentric spatial processing and its relationship with cognitive disorders.

I have read this submission. I believe that I have an appropriate level of expertise to confirm that it is of an acceptable scientific standard.

# Peer review: Referee response for version 1

**DOI:** 10.5256/f1000research.14855.r30711

**Published:** 2018-02-26

**Authors:** Sathyanarayanan V. Puthanveettil

**Affiliations:** 1 Department of Neuroscience, Scripps Research Institute, Jupiter, FL, USA

This is a beautifully written article by Miniaci and De Leonibus. Importantly this article is timely because more and more people are reported to have dementia and that both industry and academic research is struggling to produce therapeutics to cure dementia or for efficient long-term management of dementia. In this review article, Miniaci and De Leonibus discuss forms of spatial representations (allocentric and egocentric) in the brain and how these representations are altered in normal aging as well as in autism and Alzheimer's disease. The review is well written and images included are highly useful to the readers. 

 Few suggestions:

1. Revise the abstract so that it reads smoothly.
2. If the cartoons drawn (including brain images) are modified versions of previous images, earlier work should be cited directly in the figure legend. If it is adapted from a database, authors need to include links to the image.
3. It is not clear how the authors are trying to distinguish the egocentric/allocentric spatial deficits in mCI versus Alzheimer's disease.
4. Conclusion section should also need comments on spatial deficits in ASD and comparison with AD.

I have read this submission. I believe that I have an appropriate level of expertise to confirm that it is of an acceptable scientific standard.
